# Metabolic diversity in human non-small cell lung cancer

**DOI:** 10.1186/2049-3002-2-S1-P13

**Published:** 2014-05-28

**Authors:** Pei-Hsuan Chen, Ling Cai, Hyun Seok Kim, Rebecca Britt, Guanghua Xiao, Michael A White, John D Minna, Ralph J DeBerardinis

**Affiliations:** 1 Children’s Medical Center Research Institute, UT Southwestern Medical Center, Dallas, TX, USA; 2 Department of Cell Biology, UT Southwestern Medical Center, Dallas, TX, USA; 3 Hamon Center For Therapeutic Oncology, UT Southwestern Medical Center, Dallas, TX, USA; 4 Quantitative Biomedical Research Center, UT Southwestern Medical Center, Dallas, TX, USA

## Background

Cancer cells display oncogene-driven rewiring of metabolism to produce energy and macromolecules for growth. Inhibition of growth-promoting metabolic pathways may prove to be a useful therapeutic strategy in cancer. We previously identified distinct metabolic platforms that enabled cancer cells to produce macromolecular precursors from glucose and glutamine, the two most abundant nutrients. However, neither the full breadth of cancer cell metabolic diversity, nor the complement of mechanisms by which tumor mutations elicit metabolic reprogramming, are known. Because metabolic flux can be analyzed *in vitro* without any *a priori* knowledge of tumor genetics or drug sensitivity, metabolic phenotyping could produce actionable biomarkers to optimize therapy. Here we used a well-characterized panel of lung cancer cell lines to develop the most comprehensive view of cancer cell metabolism to date.

## Methods

81 non-small cell lung cancer cell lines were analyzed for a set of well-defined metabolic parameters. These cell lines were also subjected to extensive genomic, epigenetic, and gene expression analysis, and tested for sensitivity to chemotherapeutic agents and genome-wide siRNA screens. These rich data sets will streamline the establishment of novel correlations between metabolism and molecular/cell biological features. All cell lines were characterized for nutrient utilization, nutrient addiction, and focused flux assays to trace the metabolism of isotope-labeled glucose and glutamine in identical culture condition. Ongoing experiments are assessing metabolism of the same cells grown *in vivo*.

## Results

The 81 cell lines displayed surprising metabolic heterogeneity. Although all cells used glucose and glutamine to produce biosynthetic precursors, contributions of these two nutrients varied considerably, as did the pathways used. For individual isotopic labeling patterns, differences of >30-fold were observed across the panel. Unsupervised clustering produced two metabolic superfamilies differentiated by relatively glucose carbon contribution into precursor pools, as well as numerous subfamilies which may correlate to different metabolic pathways. Although KRAS-mutant cells were distributed across panel, the LKB1/KRAS co-mutation cells aggregated into the low-glucose m0 superfamily. Ongoing work will examine the ability of metabolic phenotyping to predict combinatorial mutations and vulnerabilities to drugs and siRNAs.

## Conclusion

Focused metabolic assays can produce a highly informative view of the metabolic phenotyping among large panels of cell lines. It describes an unparalleled view of the connections between genetics, drug sensitivity and cell-autonomous metabolism in NSCLC.

